# Investigating Cortical Inhibition in First-Degree Relatives and Probands in Schizophrenia

**DOI:** 10.1038/srep43629

**Published:** 2017-02-27

**Authors:** Natasha Radhu, Luis Garcia Dominguez, Tiffany A. Greenwood, Faranak Farzan, Mawahib O. Semeralul, Margaret A. Richter, James L. Kennedy, Daniel M. Blumberger, Robert Chen, Paul B. Fitzgerald, Zafiris J. Daskalakis

**Affiliations:** 1 Novartis Pharmaceuticals Canada Inc., Dorval, Quebec, Canada; 2 Temerty Centre for Therapeutic Brain Intervention, Centre for Addiction and Mental Health, University of Toronto, Toronto, Ontario, Canada; 3 Department of Psychiatry, School of Medicine, University of California, San Diego, La Jolla, California, USA; 4 Frederick W. Thompson Anxiety Disorders Centre, Sunnybrook Health Sciences Centre, University of Toronto, Toronto, Ontario, Canada; 5 Campbell Family Mental Health Research Institute, Centre for Addiction and Mental Health, University of Toronto, Toronto, Ontario Canada; 6 Division of Neurology, Krembil Research Institute, University of Toronto, Toronto, Ontario, Canada; 7 Monash Alfred Psychiatry Research Centre, The Alfred and Monash University Central Clinical School, Victoria, Australia

## Abstract

Deficits in GABAergic inhibitory neurotransmission are a reliable finding in schizophrenia (SCZ) patients. Previous studies have reported that unaffected first-degree relatives of patients with SCZ demonstrate neurophysiological abnormalities that are intermediate between probands and healthy controls. In this study, first-degree relatives of patients with SCZ and their related probands were investigated to assess frontal cortical inhibition. Long-interval cortical inhibition (LICI) was measured from the dorsolateral prefrontal cortex (DLPFC) using combined transcranial magnetic stimulation (TMS) and electroencephalography (EEG). The study presents an extended sample of 129 subjects (66 subjects have been previously reported): 19 patients with SCZ or schizoaffective disorder, 30 unaffected first-degree relatives of these SCZ patients, 13 obsessive-compulsive disorder (OCD) patients, 18 unaffected first-degree relatives of these OCD patients and 49 healthy subjects. In the DLPFC, cortical inhibition was significantly decreased in patients with SCZ compared to healthy subjects. First-degree relatives of patients with SCZ showed significantly more cortical inhibition than their SCZ probands. No differences were demonstrated between first-degree relatives of SCZ patients and healthy subjects. Taken together, these findings show that more studies are needed to establish an objective biological marker for potential diagnostic usage in severe psychiatric disorders.

SCZ is a severe psychotic disorder characterized by positive symptoms, negative symptoms and cognitive impairments^1^. OCD typically manifests in compulsive urges to perform irrational behaviors associated with the occurrence of obsessions (disturbing intrusive thoughts or impulses)^1^,^2^,^3^,^4^. There is significant overlap between SCZ and OCD vis à vis the severity of their psychopathology, affected brain areas, clinical symptom profile, and pharmacotherapy^5^.

In psychiatry, there are no objective laboratory tests to inform diagnoses and monitor response to treatment. Endophenotypes are genetically determined phenotypes that may be part of a complex disease and facilitate the development of etiologic rather than symptom-based diagnostic methods. They help to advance our understanding of the genetic mechanisms underlying psychiatric disorders^6^,^7^. Unaffected first-degree relatives of SCZ patients are ideal candidates as they share degrees of genetic vulnerability and are free from antipsychotic treatment and psychopathology^8^.

Deficits in GABAergic inhibitory neurotransmission have been a reliable finding in SCZ across multi-modal investigative approaches. These deficits may be due to an imbalance between cortical excitation and inhibition of the cortex^9^. For example, Benes *et al*. reported a decreased density of non-pyramidal cells in anterior cingulate layers II-VI and in prefrontal cortex layer II in SCZ^10^. Akbarian *et al*. found reduced messenger RNA (involved in the synthesis of GABA) in the dorsolateral prefrontal cortex (DLPFC) of SCZ patients^11^. Previous studies have shown that SCZ patients exhibit deficits in GABAergic inhibition using transcranial magnetic stimulation (TMS)^12^,^13^,^14^,^15^,^16^,^17^,^18^,^19^,^20^, limited to the motor cortex. Limited studies have investigated first-degree relatives of SCZ patients using TMS paradigms. Saka *et al*.^21^, evaluated TMS measures of inhibition in unaffected first-degree relatives of SCZ patients compared to healthy subjects (no proband group was assessed). They found that 25% of first-degree relatives lacked transcallosal inhibition and showed psychosis-proneness relative to healthy controls. Taken together, these findings show that more studies are needed to establish an objective biological marker for potential diagnostic usage in severe psychiatric disorders.

TMS combined with electroencephalography (EEG) is a powerful tool for investigating cortical mechanisms and networks of frontal brain areas. Recent technical advances have enabled the concurrent recording of TMS and EEG. Using this approach, GABA_B_ receptor-mediated inhibitory neurophysiological mechanisms can be measured through a paired-pulse paradigm, long-interval cortical inhibition (LICI). Farzan *et al*. have demonstrated using TMS-EEG that LICI of gamma oscillations were selectively impaired in the DLPFC of patients with SCZ compared to both healthy subjects and similarly treated patients with bipolar disorder^22^. Patients with bipolar disorder were similar to patients with SCZ in relation to severity of symptoms, illness duration, and history of psychosis. In a recent study, it was also found that frontal LICI was significantly reduced in SCZ patients, compared to OCD patients and healthy subjects, also showing no effect of antipsychotic medication^23^. These findings suggest that LICI abnormalities may be specific to SCZ and are not part of a generalized deficit associated with severe psychopathology. TMS evoked potentials are thought to reflect changes in cortical excitability potentially related to longer lasting underlying inhibitory post-synaptic potentials and excitatory post-synaptic potentials rather than neural firing.

The objective of the present study was to evaluate frontal GABA_B_-mediated cortical inhibition in patients with SCZ, patients with OCD patients, both of their unaffected first-degree relatives compared to healthy subjects. Based on previous studies^22^,^23^, we hypothesized that frontal inhibition would be significantly reduced in patients with SCZ. We also hypothesized that frontal inhibition in first-degree relatives of SCZ would be intermediate of healthy subjects and their related probands. Lastly, it was postulated that inhibitory impairments would not be shown in OCD patients and their first-degree relatives.

## Materials and Methods

The study assessed 129 subjects, Table 1 includes the demographic information for all subjects. The data includes a subset of subjects that were previously published (13 SCZ patients, 7 OCD patients and 43 healthy subjects were not overlapping)^23^. All subjects gave their written informed consent and the protocol was approved by the Centre for Addiction and Mental Health (CAMH) in accordance with the Declaration of Helsinki. The 66 subjects that have been previously reported and were recruited from CAMH advertisements and patient research registries. The Structured Clinical Interview for the Diagnostic and Statistical Manual for Mental Disorders (DSM)-IV^1^ confirmed diagnosis of SCZ, schizoaffective disorder or OCD. Medications and diagnostic information of SCZ and OCD patients are shown in Tables 2, 3 and 4. In healthy subjects, psychopathology was ruled out by the Structured Clinical Interview for DSM-IV and healthy subjects were only included in the study if they had no first-degree relative diagnosed with a psychiatric disorder. Healthy subjects and all first-degree relatives of probands were administered the Family Interview for Genetic Studies^24^. Relatives of probands had no psychopathology in the last 2 years as ruled out through the Structured Clinical Interview for DSM-IV and were not confounded by the use of psychotropic medication. First-degree relatives were recruited through public advertisements (6 family members of SCZ patient, 3 related SCZ probands and 8 family members of OCD patients, 5 related OCD probands). The remaining first-degree relatives were recruited from referrals from their related probands that were enrolled in the study. Participation of at least one first-degree relative of a proband was a requirement for this study; the proband and either one biological parent or one full sibling was necessary for the neurophysiological assessments. The Schizotypal Personality Questionnaire (SPQ)^25^ was used for evaluating psychopathology in first-degree relatives of SCZ patients. The 24-construct Brief Psychiatric Rating Scale (BPRS) was used for evaluating psychopathology in SCZ patients^26^.

The Yale-Brown Obsessive Compulsive Scale (YBOCS) was used for evaluating psychopathology in OCD patients^27^,^28^. Exclusion criteria included: (1) DSM-IV criteria for substance abuse or dependence in the last 6 months, except nicotine; (2) unstable medical or neurological illness; (3) suicidal ideation; (4) pregnancy; (5) positive urine toxicology screen for drugs of abuse; (6) magnetic material or other conditions that would preclude the magnetic resonance image (MRI) or TMS-EEG; (7) clinically significant claustrophobia. The exclusion criteria established by international safety standards for TMS were followed^29^.

### Data Recording

#### Transcranial Magnetic Stimulation

Monophasic TMS pulses were administered using a 7 cm figure-of-eight coil, and two Magstim 200 stimulators (Magstim Company Ltd, UK) connected via a Bistim module. TMS was administered over the left motor cortex and DLPFC in separate blocks. One hundred TMS stimuli were delivered per-condition (paired and single-pulse) every 5 seconds. LICI was obtained at the 100 ms interstimulus interval^30^. The intensity of both the conditioning and test stimuli were set to elicit an average motor evoked potential of 1 mV peak-to-peak (suprathreshold stimulation).

#### Localization of the Motor Cortex

The TMS coil was placed at the optimal position for eliciting motor evoked potentials from the right abductor pollicis brevis muscle, which corresponded to electrodes FC3 and C3. Electromyography was captured by placing two disposable disc electrodes over the right abductor pollicis brevis muscle and motor evoked potentials were filtered (band-pass 2 to 5 kHz), digitized at 5 kHz (Micro 1401, Cambridge Electronics Design, Cambridge, UK).

#### Localization of DLPFC

Localization of DLPFC was achieved through neuronavigation techniques using the MINIBIRD system (Ascension Technologies) and MRIcro/registration software using a T1-weighted MRI scan obtained for each subject with seven fiducial markers in place^22^,^31^. Stimulation was directed at the junction of the middle and anterior one-third of the middle frontal gyrus (Talairach coordinates (x, y, z) = −50, 30, 36) corresponding with posterior regions of Brodmann area 9, which overlap with the superior section of Brodmann area 46.

#### EEG Recording and Pre-Processing

To evaluate TMS-induced cortical evoked potentials, EEG was recorded concurrently with electromyography. EEG was acquired through a 64-channel Synamps 2 EEG system. A 64-channel EEG cap was used to record the cortical signals, and four electrodes were placed on the outer side of each eye, and above and below the left eye to closely monitor eye movement artifacts. All electrodes were referenced to an electrode positioned posterior to Cz electrode. EEG signals were recorded DC and with a low pass filter of 100 Hz at a 20 kHz sampling rate, to avoid saturation of the amplifiers and to minimize TMS-related artifact^31^,^32^.

EEG recordings were down-sampled to 1000 Hz and epoched from −1000 ms to 2000 ms after the test TMS pulse. In both, the single and paired-pulse conditions, the data from −100 ms to 10 ms was fully removed (where 0 correspond to the test TMS pulse). This step removes the test-pulse TMS from both of the single-pulse and paired-pulse conditions and also the conditioning TMS pulse from the paired-pulse condition. Traces were visually inspected for artifacts in order to eliminate trials and channels highly contaminated by noise (muscle activity, 60 Hz noise, and movement-related activity as well as electrode artifacts). Two rounds of independent component analysis were subsequently applied. The first round was to minimize and remove the typical TMS-related decay artifact. Following this, a bandpass FIR filter was applied from 1 to 55 Hz and a second round of independent component analysis was computed to remove eye movement-related artifacts (blinks and movements) and muscle components.

## Analyses

### Post-Processing Analyses

Time-frequency decomposition was obtained using the Event-Related Spectral Perturbation (ERSP) analysis in EEGLab. Specifically the analysis was wavelet based, using a cycle of the complex Morlet wavelet across frequencies 2–50 Hz. The ERSP was computed independently for the single-pulse and paired-pulse conditions. The analysis is expressed in decibels of spectral power (μV^2^/Hz) after subtracting the log baseline to the whole trial. In two previous studies, we have shown that inhibition can be evaluated as the difference between the single and paired-pulse conditions, using a measure of amplitude of the evoked activity^23^,^33^. It has been demonstrated previously that the test pulse can be masked by the excitatory effect of the conditioning pulse. We corrected for this by subtracting the paired pulse from the single pulse lined up to the conditioning pulse. In this study, the measure of amplitude is the power of the wavelet decomposition. Nine electrodes (F1, Fz, F2, FC1, FCz, FC2, C1, Cz, C2) were retained for the analysis of inhibition (DLPFC and motor cortex). Supplementary Figure 1 demonstrates both the paired-pulse and single-pulse data.

### Calculating Inhibition by Subject

In this study, we computed the difference in the evoked power of the two conditions; we also computed a number of paired surrogate conditions made of sets of randomly selected trials, without replacement from the pool of trials of the two conditions. The surrogate conditions served as a baseline or null hypothesis for the case of no inhibition, i.e. no difference between the powers of conditions. The power differences were extracted from the original conditions as well as from the surrogate ones to consist of a set of values within the voxels in the time-frequency-electrode space for each subject. From this “landscape” of values over the time-frequency-electrode space, a threshold (p-value) was chosen to label each voxel as inhibited = 1 or not-inhibited = 0. A voxel received a value of “1” if its value is greater than 99% of the values in the same voxel in the null distribution, otherwise the value was set at “0”. The number of randomization in the null distribution was set to 500. Inhibition was then evaluated by counting the 1’s that forms a cluster in this time-frequency-electrode domain. A voxel belongs to a cluster if it has a value of 1, and has at least a neighbour in time, frequency and electrode that is also in the cluster^33^. Thus, for each subject an index of inhibition is defined as the total sum of significant values in the largest cluster. The cluster is only considered in a time domain from the moment of the test pulse to 500 ms after, and frequencies from 2–50 Hz. If the analysis is restricted to the gamma band we sum only over the range: 30–50 Hz. The size of the largest cluster of significant values (or index of inhibition) is a way to capture the degree of inhibition at the subject level. The specific applications of the cluster mass test are derived from two publications^23^,^33^.

### Between-Group Analyses

After calculating inhibition by subject, we compared groups by a t-test with pooled variance. In all comparisons, the two-tailed analyses are reported. However, based on our previous finding^23^, one analysis was single-tailed when we compared healthy subjects to SCZ patients in overall (2–50 Hz) and gamma (30–50 Hz) inhibition. The null hypothesis was that the control group is not significantly more inhibited than the SCZ group.

**Figure 1:**
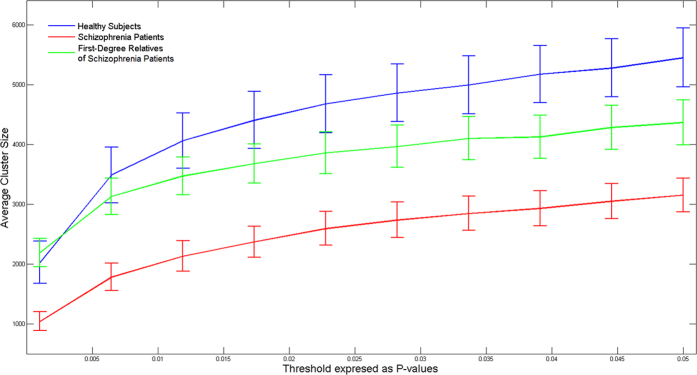
The index of frontal inhibition from a cluster analysis at different thresholds of p-values in healthy controls, first-degree relatives of schizophrenia patients and their related probands. A cluster analysis was performed for each group by sampling a subset 19 of subjects with replacement. The procedure was repeated 2000 times for each threshold. Error bars are standard error of the mean.

**Figure 2:**
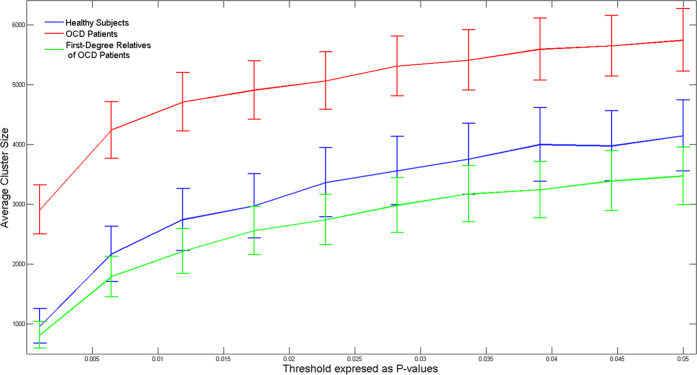
The index of frontal inhibition from a cluster analysis at different thresholds of p-values in healthy controls, first-degree relatives of obsessive-compulsive disorder patients and their related probands. A cluster analysis was performed for each group by sampling a subset 13 of subjects with replacement. The procedure was repeated 2000 times for each threshold. Error bars are standard error of the mean.

Figure 1 depicts differences in inhibition evaluated non- parametrically for patients with SCZ, their first-degree relatives and healthy subjects, as follows. A value of inhibition was obtained from a subset of 19 subjects (that were chosen with replacement) as the largest cluster size with significant values resulting from voxel-by-voxel paired t-test between the single and paired-pulse stimulation. Since inhibition corresponds to the power of the single pulse being larger than that of the paired-pulse, the analysis was single-tailed^23^,^33^. This analysis was repeated 2000 times, selecting a different pool of 19 subjects from the same group. We replicated the same analysis for healthy controls, OCD patients and their unaffected first-degree relatives (Fig. 2).

### Stratification of Age in First-Degree Relatives of Schizophrenia Patients

DLPFC inhibition (overall and gamma frequency bands) was compared between young (<50 years) first-degree relatives of SCZ patients and older (>50 years) first-degree relatives of SCZ patients to determine whether there were effects of age. An independent samples t-test was used.

### Evaluating Clinical Severity in First-Degree Relatives of Schizophrenia

A Spearman’s rho correlation analysis was performed between the SPQ and the index of frontal inhibition (overall and gamma frequency bands) for the first-degree relatives of SCZ group.

### Assessing Clinical Severity and Effects of Medication Analyses

A Spearman’s rho correlation analysis was performed between the BPRS and the index of frontal inhibition (overall and gamma frequency bands) for each SCZ patients. A Spearman’s rho correlation analysis was performed between the YBOCS and the index of frontal inhibition (overall and gamma frequency bands) for each OCD patient. A Spearman’s rho was also conducted with antipsychotic medication dosage expressed as chlorpromazine equivalents^34^,^35^,^36^,^37^.

DLPFC inhibition (overall and gamma frequency bands) was compared with antidepressant-treated patients and patients not treated with antidepressants in both SCZ and OCD patient groups. An independent samples t-test was used. DLPFC inhibition (overall and gamma frequency bands) was compared with benzodiazepine-treated patients and patients who were not treated with benzodiazepines in both SCZ and OCD patient groups. An independent samples t-test was used.

### The Heritability of Inhibition in Schizophrenia

Variance components methods in SOLAR v.4.3.1 were used to estimate the narrow sense heritability for overall inhibition of the DLPFC, defined as the phenotypic variance explained by additive genetic factors^38^. Normalized trait values were used for all analyses, and age and sex were screened as covariates and found to be not significant (p > 0.05). In first-degree relatives of SCZ patients, there were no differences in ethnicity between Caucasians and non-Caucasians in frontal overall inhibition (p = 0.39) and frontal gamma inhibition (p = 0.85). In SCZ patients, there were no difference in ethnicity between Caucasians and non-Caucasians in frontal overall inhibition (p = 0.25) and frontal gamma inhibition (p = 0.58), thus, ethnicity was not used a covariate. Corrections were made for ascertainment bias, since the families were recruited through the identification of a proband with SCZ and are thus not representative of the general population^39^. The overall measure was the only measure selected for heritability as significant group differences were observed between SCZ probands and controls and between SCZ probands and relatives.

## Results

### Frontal Overall (2–50 Hz) Inhibition

**Figure 3:**
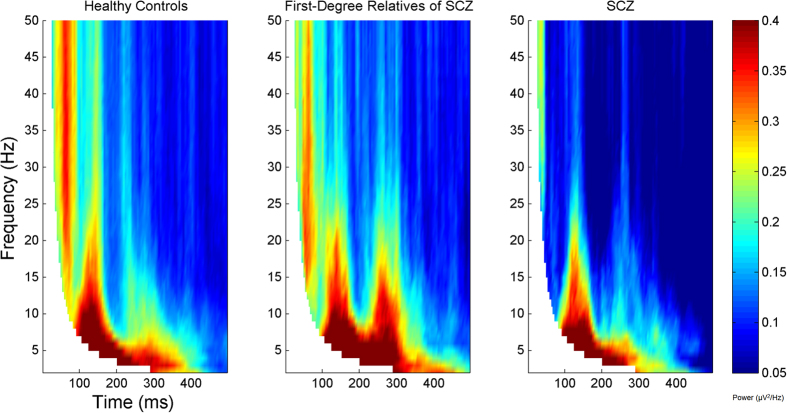
The frequency of significant values for each group, summarized from subject data, on each voxel for all the nine central electrodes (F1, Fz, F2, FC1, FCz, FC2, C1, Cz, C2). The threshold for significance was chosen to be p < 0.01. Each graph corresponds to healthy subjects, first-degree relatives of SCZ patients, and their related probands. The left dorsolateral prefrontal cortex was stimulated. Values are masked over the bottom left area (denoted in white) indicating that those specific windows of the wavelet analysis contains points from the pre-stimulus interval.

**Figure 4:**
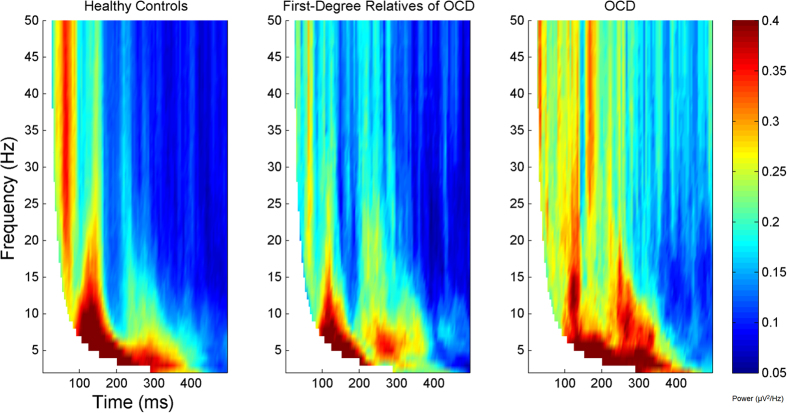
The frequency of significant values for each group, summarized from subject data, on each voxel for all the nine central electrodes (F1, Fz, F2, FC1, FCz, FC2, C1, Cz, C2). The threshold for significance was chosen to be p < 0.01. Each graph corresponds to healthy subjects, first-degree relatives of obsessive-compulsive disorder patients, and their related probands. The left dorsolateral prefrontal cortex was stimulated. Values are masked over the bottom left area (denoted in white) indicating that those specific windows of the wavelet analysis contains points from the pre-stimulus interval.

Frontal inhibition was significantly greater in healthy subjects compared to subjects with SCZ (t = 1.89, df = 66, p = 0.032). First-degree relatives of SCZ patients showed significantly more inhibition than their SCZ probands (t = 2.24, df = 47, p = 0.03). No differences were demonstrated between first-degree relatives of SCZ and healthy subjects (t = −0.39, df = 77, p = 0.69). Figure 1 shows that the pattern of frontal inhibition. This analysis showed that the pattern of inhibition was: healthy subjects >first-degree relatives of SCZ > SCZ probands, over a wide range of p-value thresholds and was independent of the specific threshold chosen. A heritability estimate of 0.41 ± 0.43 (p = 0.16) was observed for overall inhibition. No differences were found between healthy subjects compared to OCD patients (t = −0.99, df = 60, p = 0.32) and their first-degree relatives (t = −0.06, df = 65, p = 0.95). Lastly, no inhibition differences were found between OCD patients and their first-degree relatives (t = −0.74, df = 29, p = 0.46). Figures 3 and 4 depict group inhibition over the time-frequency domain evaluated non-parametrically.

### Frontal Gamma (30–50 Hz) Inhibition

Gamma inhibition was significantly lower in SCZ compared to healthy subjects (t = 2.22, df = 66, p = 0.015). No significant differences were found between healthy subjects and first-degree relatives of SCZ (t = 0.27, df = 77, p = 0.79). When comparing first-degree relatives of SCZ to their probands, non-significant differences in gamma inhibition were shown (t = 1.69, df = 47, p = 0.098). No significant differences were found between healthy controls compared to OCD (t = −1.24, df = 60, p = 0.22) and when compared to first-degree relatives of OCD (t = −0.13, df = 65, p = 0.89). Lastly, no significant differences were found between first-degree relatives of OCD compared to their OCD probands (t = −0.82, df = 29, p = 0.42).

### Motor Cortex Overall (2–50 Hz) Inhibition

No significant inhibitory differences were found between healthy subjects and SCZ patients (t = 0.22, df = 66, p = 0.41) or healthy subjects as compared to their first-degree relatives (t = −0.44, df = 77, p = 0.66). No significant differences were found between SCZ patients and their first-degree relatives (t = 0.57, df = 47, p = 0.57). No differences were found between healthy subjects compared to OCD patients (t = −0.29, df = 60, p = 0.78) and when compared to their first-degree relatives (t = 0.24, df = 65, p = 0.81). No significant differences were found between OCD patients and their first-degree relatives (t = −0.58, df = 29, p = 0.57).

### Motor Cortex Gamma (30–50 Hz) Inhibition

No significant inhibitory differences were found between healthy subjects and SCZ patients (t = 0.04, df = 66, p = 0.49) or healthy subjects as compared to their first-degree relatives (t = 0.22, df = 77, p = 0.83). No significant differences were found between SCZ patients and their first-degree relatives (t = −0.16, df = 47, p = 0.87). No differences were found between healthy subjects compared to OCD patients (t = 0.15, df = 60, p = 0.88) and when compared to their first-degree relatives (t = 0.63, df = 65, p = 0.53). No significant differences were found between OCD patients and their first-degree relatives (t = −0.53, df = 29, p = 0.60).

### Stratification of Age in First-Degree Relatives of Schizophrenia Patients

No significant differences were found when comparing younger (n = 9) (<50 years) to older (n = 21) (>50 years) first-degree relatives of SCZ patients in frontal overall inhibition (p = 0.48) and frontal gamma inhibition (p = 0.66).

### Clinical Severity Analysis in First-Degree Relatives of Schizophrenia Patients

In first-degree relatives of SCZ patients, no significant relationship was found between the SPQ (total score) and frontal overall inhibition (Spearman’s rho = 0.27, p = 0.92). In first-degree relatives of SCZ, no significant relationship was found between the SPQ (total score) and frontal gamma inhibition (Spearman’s rho = 0.14, p = 0.76).

### Effect of Antipsychotic Medications and Anti-depressant Medications

No significant correlation between overall inhibition and chlorpromazine equivalents was shown (Spearman’s rho = 0.13, p = 0.71) and no relationship was found between frontal gamma inhibition and chlorpromazine equivalents (Spearman’s rho = 0.0, p = 0.50).

In the DLPFC, no significant differences were found between antidepressant-treated OCD patients (n = 7) and unmedicated OCD patients (n = 6) in overall inhibition (p = 0.15) and gamma inhibition (p = 0.32). In the DLPFC, no significant differences were found between antidepressant-treated SCZ patients (n = 4) and SCZ patients who were not treated with antidepressants (n = 15) in overall frontal inhibition (p = 0.89) and frontal gamma inhibition (p = 0.92). In the DLPFC, no significant differences were found between OCD patients who were treated with SSRIs (n = 4) and SCZ patients who were treated with SSRIs (n = 2) in overall frontal inhibition (p = 0.40) and gamma inhibition (p = 0.31).

Lastly, in the DLPFC, no significant differences were found between benzodiazepine-treated OCD patients (n = 4) and OCD patients (n = 9) who were not treated with benzodiazepines in overall frontal inhibition (p = 0.78) and frontal gamma inhibition (p = 0.95). We also found no significant differences between benzodiazepine-treated SCZ patients (n = 4) and SCZ patients (n = 15) who were not treated with benzodiazepines in overall frontal inhibition (p = 0.31) and frontal gamma inhibition (p = 0.66).

### Clinical Severity in Schizophrenia Patients

No significant relationship was found between the BPRS and overall frontal inhibition, (Spearman’s rho = −0.17, p = 0.24). No significant relationship was found between the BPRS and frontal gamma inhibition (Spearman’s rho = −0.14, p = 0.29).

### Clinical Severity in OCD Patients

No significant relationship was found between the YBOCS and overall frontal inhibition, (Spearman’s rho = 0.47, p = 0.95). No significant relationship was found between the YBOCS and frontal gamma inhibition (Spearman’s rho = 0.23, p = 0.77).

### Classification Analysis

The receiver operating characteristic (ROC) curve analysis was completed to quantify the cluster size for both overall inhibition and gamma inhibition as a classifier based on the sensitivity and specificity. Healthy and OCD patients were combined to compare to SCZ. The reported values were the area under the ROC curve indexing a combined sensitivity and specificity. A significant area greater than 0.50 indicates that the value is better than a guess at random (i.e. flipping a coin) and a perfect test is 1.0. Youden’s J statistic was reported and demonstrates the peak of the ROC curve to index specificity and sensitivity seperately^40^. The classification analysis showed a significant area under the ROC curve for overall inhibition (0.65, p = 0.047) and gamma inhibition (0.74, p = 0.002). The sensitivity was 0.63 and specificity was 0.66 for overall inhibition. The sensitivity was 0.89 and specificity was 0.60 for gamma inhibition.

## Discussion

We found that first-degree relatives of SCZ patients showed an intermediate pattern of frontal inhibition compared to their related probands and healthy controls. Significant differences were found in frontal overall inhibition between SCZ patients and their unaffected first-degree relatives. Significant deficits were shown in frontal overall and gamma inhibition in patients with SCZ compared to healthy subjects. A sensitivity of 89% for frontal gamma inhibition was found.

### Frontal Inhibition in First-Degree Relatives of Schizophrenia Probands

We demonstrated that first-degree relatives of SCZ had significant differences in overall inhibition when compared to their related probands. First-degree relatives of SCZ were not significantly different from healthy subjects. The pattern of frontal inhibition in first-degree relatives of SCZ was intermediate between their related probands and healthy controls (Fig. 1).

Data from biological relatives of probands are important for assessing disease-related effects in a complex disorder like SCZ. The Consortium of Genetics in Schizophrenia (COGS) has investigated several neurophysiological measures as potential endophenotypes as a means for understanding the genetic determinants of SCZ^24^. Greenwood *et al*. demonstrated that P50 suppression shows a low heritability of 0.10 that was not significant in a sample of 183 nuclear families^41^. Furthermore, for the antisaccade task for eye movements, moderate to strong heritability of 0.42 was found^41^ and a modest heritability of 0.32 for prepulse inhibition was shown^41^. Hasenkamp *et al*. demonstrated moderate heritability of 0.45 for prepulse inhibition at the 60 ms interstimulus interval and a trending heritability of 0.33 at the 120 ms interstimulus interval^42^. Identification of biomarkers are needed to facilitate diagnosis in the future and to facilitate the identification of genes contributing to SCZ susceptibility.

### Intermediate Phenotypes in Schizophrenia

Currently, no objective measures exist to inform psychiatric diagnoses as the clinical interview dominates the diagnostic approach. Psychiatric disorders are complex, no specific constellation of genes or environmental conditions characterize a large subset of ill individuals^43^. A gene linked with behavioral abnormalities may be more strongly associated with a measure of brain function related to SCZ and genetic risk for SCZ, in the absence of clinical presentation^44^,^45^. Specificity and heritability do not both have to be present to develop a biomarker for diagnostic purposes. As an example, blood glucose levels are used to diagnose diabetes while blood glucose heritability has been shown to be low^46^. Taken together, frontal inhibition may be further explored further as an objective measure in clinical settings for SCZ.

### Sensitivity in Schizophrenia

In this study, we found a sensitivity of 89% for gamma inhibition, also known as the true-positive rate. This finding suggests that gamma inhibition can accurately identify SCZ at a rate of 89%, providing a positive test result. Gamma inhibition has been shown to be a more informative measure based on the classification analyses. Gamma inhibition of the DLPFC may be explored further in SCZ based on the high sensitivity results.

### Frontal Inhibitory Deficits in Schizophrenia

GABAergic deficits have been found in SCZ based on post-mortem and animal studies showing reduced expression of pre- and postsynaptic markers of GABAergic neurotransmission in subpopulations of GABAergic interneurons in the prefrontal cortex^47^,^48^,^49^. Recent work^50^ suggests that SCZ patients had significantly lower GABA/creatine ratios in the medial prefrontal cortex using magnetic resonance spectroscopy. These findings are consistent with postmortem SCZ studies demonstrating diminished GABA production based on decreased levels of mRNA encoding for glutamate decarboxylase67 (GAD67), an enzyme that facilitates GABA synthesis from glutamate^51^,^52^,^53^,^54^. As a result of the above mentioned findings, excessive excitability in the cortex may result in aberrant neuronal activation that may lead to the disorganized behavior and impulsivity, demonstrated in SCZ^55^,^56^.

### Limitations

The main challenge of family-based studies is recruitment. Larger samples of first-degree relatives are needed to increase power and address the heritability question. In future, multi-center research trials are needed to develop TMS-EEG as a neurophysiological method for use in clinical settings. Furthermore, patients with SCZ were treated with a variety of antipsychotic medications and other psychotropic medications and were chronically ill, which may have effects on neural oscillations. Future studies should recruit unmedicated patients to assess these medication effects.

## Conclusions

This study shows that impairments in frontal inhibition are specific to the pathophysiology of SCZ and may have the potential for use in diagnosis. The search for liability genes for complex disorders such as SCZ may be aided by identifying endophenotypes and relating these genes to cortical inhibition.

## Additional Information

**How to cite this article:** Radhu, N. *et al*. Investigating Cortical Inhibition in First-Degree Relatives and Probands in Schizophrenia. *Sci. Rep.* **7**, 43629; doi: 10.1038/srep43629 (2017).

**Publisher's note:** Springer Nature remains neutral with regard to jurisdictional claims in published maps and institutional affiliations.

## Supplementary Material

Supplementary Figure 1

## Figures and Tables

**Table 1:** Demographics of all study participants.

Group	Healthy Controls	Schizophrenia Schizoaffective Patients	First-Degree Relatives of Schizophrenia	Obsessive-Compulsive Disorder (OCD) Patients	First-Degree Relatives of OCD
Sample size	49	19	30	13	18
Age	33.4	30.2	53.8	28.9	41.9
Females/Males	25/24	9/10	17/13	9/4	12/6
Handedness	42 Right	16 Right	26 Right	11 Right	16 Right
	4 Left	2 Left	4 Left	2 Left	1 Left
	3 Ambidextrous	1 Ambidextrous			1 Ambidextrous
1 mV intensity mean (SD)	69.02 (13.06)	59.84 (10.69)	60.37(7.41)	56.15 (9.81)	59.39 (10.75)
Number of family members	NA	NA	18 families	NA	13 families
			1.61 members		1.38 members
Parents	NA	NA	23	NA	9
			13 mothers		5 mothers
			10 fathers		4 fathers
Siblings	NA	NA	7	NA	9
			4 sisters		7 sisters
			3 brothers		2 brothers

**Table 2:** Description of the Psychotropic Medications for Schizophrenia Patients Displayed as Number of Subjects/Dose(s).

Patients with Schizophrenia Medication Details		
CLASS	MEDICATION	# OF SUBJECTS/DOSE(S) in mg
ANTIPSYCHOTICS		
Second Generation	Clozapine	n = 9: 150 (1), 200 (1), 250 (2), 300 (2), 350 (1), 400 (1) 475 (1)
	Olanzapine	n = 2: 7.5, 22.5
	Paliperidone	n = 1: 150/4 weeks
	Quetiapine	n = 1: 300
	Risperidone	n = 3: 2 (2), 3
	Risperidone Injection	n = 2: 50/2 weeks, 75/4 weeks
	Ziprasidone	n = 1: 60
Dibenzoxazepines	Loxapine	n = 1: 30
Third Generation	Aripiprazole	n = 2: 20, 30
ANTIDEPRESSANTS		
Selective serotonin re-uptake inhibitors (SSRIs)	Citalopram	n = 2: 40 (2)
Serotonin–norepinephrine reuptake inhibitors (SNRIs)	Desvenlafaxine	n = 1: 50
Norepinephrine-dopamine reuptake inhibitor (NDRIs)	Bupropion SR	n = 1: 150
MOOD STABILIZERS		
	Divalproex Sodium	n = 1: 500
	Lamotrigine	n = 1: 100
	Topiramate	n = 1: 200
BENZODIAZEPINES		
	Clonazepam	n = 2: 0.5, 1
	Clonazepam prn	n = 1: 0.25
	Lorazepam prn	n = 1: 2
OTHERS		
	Benzatropine	n = 1: 2

**Table 3:** Patients with Obsessive-Compulsive Disorder Medication Details.

Patients with Obsessive-Compulsive Disorder Medication Details		
CLASS	MEDICATION	# OF SUBJECTS/DOSE(S) in mg
ANTIDEPRESSANTS		
Selective serotonin re-uptake inhibitors (SSRIs)	Escitalopram	n = 1: 50
	Fluoxetine	n = 2: 20, 80
	Sertraline	n = 1: 250
Serotonin–norepinephrine reuptake inhibitors (SNRIs)	Duloxetine	n = 1: 60
Tricyclic antidepressants (TCAs)	Clomipramine	n = 4: 50, 250 (3)
Norepinephrine reuptake inhibitor (NRIs)	Atomoxetine	n = 1: 80
ANTIPSYCHOTICS		
	Loxapine	n = 1: 25
	Olanzapine	n = 1: 20
	Aripiprazole	n = 1: 2
MOOD STABILIZERS		
	Divalproex Sodium	n = 1: 750
BENZODIAZEPINES		
	Clonazepam	n = 1: 0.5
	Clonazepam prn	n = 1: 0.5
	Diazepam prn	n = 1: 4
	Temazepam	n = 1: 30

**Table 4:** Diagnostic Information for Schizophrenia and Obsessive-Compulsive Disorder patients.

Schizophrenia (N = 19)		
	Number of Subjects	%
Schizophrenia (paranoid type)	14	73.68
Schizoaffective (bipolar type)	5	26.32
Current Comorbidities	Number of Subjects	%
Major Depressive Disorder	1	5.26
Post-Traumatic Stress Disorder	1	5.26
Panic Disorder without Agoraphobia	1	5.26
**Obsessive-Compulsive Disorder (N** = **13)**		
Current Comorbidities	Number of Subjects	%
Social Phobia	4	30.77
Panic Disorder without Agoraphobia	2	15.38
Generalized Anxiety Disorder	4	30.77
Major Depressive Disorder	1	7.69
Attention Deficit Hyperactivity Disorder	1	7.69
