# Supplementary material for: Investigating Cortical Inhibition in First-Degree Relatives and Probands in Schizophrenia
Source: Sci Rep. 2017 Feb 27;7:43629. doi: 10.1038/srep43629 (PMC5378912; doi:10.1038/srep43629)
Supplement: Supplementary Figure 1 [file srep43629-s1.pdf]

Supplementary Figure 1

This figure demonstrates the averaged EEG signal across nine electrodes (F1, Fz, F2, FC1, FCz, FC2, C1, Cz, C2) for all healthy controls. This figure includes both paired-pulse (red) and single-pulse (blue) data. The discontinuity corresponds to the segment removed to mask the conditioning pulse as discussed in the article.

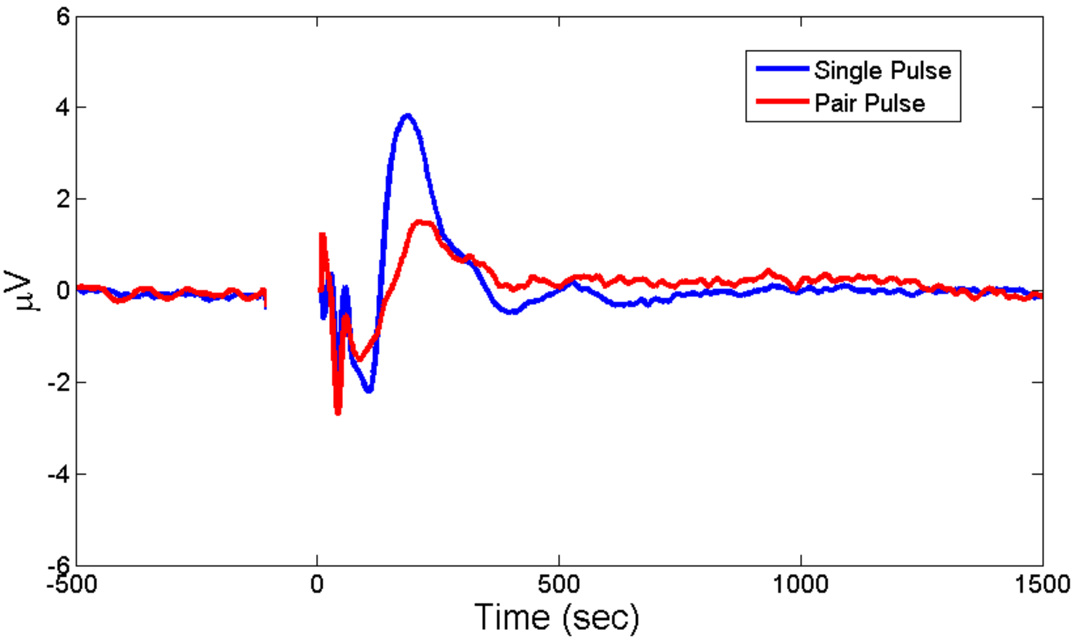

Investigating Cortical Inhibition in First-Degree Relatives and Probands in Schizophrenia

Natasha Radhu, PhD
Luis Garcia Dominguez, PhD
Tiffany A. Greenwood, PhD
Faranak Farzan, PhD
Mawahib O. Semeralul, MSc
Margaret A. Richter, MD, FRCP(C)
James L. Kennedy, MD, FRCP(C)
Daniel M. Blumberger, MD, MSc, FRCP(C)
Robert Chen, MA, MBBChir, MSc, FRCP(C)
Paul B. Fitzgerald, MBBS, MPM, PhD, FRANZCP
Zafiris J. Daskalakis, MD, PhD, FRCP(C)
